# IL-33 receptor (ST2) deficiency downregulates myeloid precursors, inflammatory NK and dendritic cells in early phase of sepsis

**DOI:** 10.1186/s12929-018-0455-z

**Published:** 2018-07-12

**Authors:** Zivan M. Babic, Filip Z. Zunic, Jelena M. Pantic, Gordana D. Radosavljevic, Ivan P. Jovanovic, Nebojsa N. Arsenijevic, Miodrag L. Lukic

**Affiliations:** 1 Department of Surgery, Faculty of Medical Sciences, University of Kragujevac, Kragujevac, Serbia; 2 Center for Molecular Medicine and Stem Cell Research, Faculty of Medical Sciences, University of Kragujevac, Svetozara Markovica 69, Kragujevac, 34000 Serbia

**Keywords:** ST2, IL-33, Sepsis, Myeloid precursors, NK cells, Dendritic cells

## Abstract

**Background:**

Sepsis is a life-threatening disease mediated by profound disturbances in systemic inflammatory response to infection. IL-33 is multifunctional regulator of numerous aspects of innate and adaptive immune response. The aim of this article was to further evaluate the role of IL-33 receptor (ST2) in different pathways of innate immunity during early polymicrobial sepsis.

**Methods:**

Polymicrobial sepsis was induced using cecal ligation and puncture (CLP) model in ST2 deficient (ST2^−/−^) and wild type BALB/c mice. Peritoneal and spleen cells were isolated for further phenotyping. Apoptosis was determined by immunohistochemistry and flow cytometry.

**Results:**

Deletion of ST2 leads to increased susceptibility to early manifestations of sepsis as evaluated by clinical signs and survival. These are accompanied by decrease in the total number of neutrophils, eosinophils and mast cells in peritoneal cavity 12 h after CLP. In early sepsis there was also low number of precursors of myeloid cells in particular CD11b^+^Ly6G^+^Ly6C^low^ cells in spleen of ST2^−/−^ mice. Although the number of NK cells in the spleen was similar, there were significant differences in the presence of inflammatory IFN-γ and IL-17 producing NK cells. Further, ST2 deletion affects the phenotype and maturation of dendritic cell in sepsis. The total number of dendritic cells in the spleen was lower as well as IL-12 expressing dendritic cells. Finally, there was higher frequency of active caspase-3 positive and early apoptotic cells, in particular CD11c positive cells, in spleen of septic ST2^−/−^ mice.

**Conclusion:**

Taken together, our data provide the evidence that ST2 deficiency in early phase of sepsis downregulates myeloid precursors, inflammatory NK and dendritic cells.

## Background

Sepsis is a life-threatening condition originating from systemic inflammatory response to acute infection followed by tissues and organs injury [1]. In sepsis, the host response triggered by the invasion of microorganism includes pro- and anti-inflammatory response, occurring early and almost simultaneously [2–4]. The initial hyperinflammatory phase following the recognition of the invading pathogen is rapidly replaced by the long term immunosuppression and subsequent disease aggravation and prolonged sensitivity to superinfections [5]. There is plethora of evidences that numerous immune cells and soluble mediators of inflammation participate in sepsis pathogenesis.

IL-33 is a multifunctional cytokine that participates in various inflammatory responses. It exerts its biological effects via IL-33 receptor containing ST2 molecule and IL-1 receptor accessory protein (IL-1RAcP) expressed on various immune and nonimmune cells (reviewed in [6]). Our previously published data revealed that IL-33/ST2 axis plays an important role in different autoimmune/inflammatory and malignant diseases by exerting multiple effects on both innate and adaptive immunity [7–12]. IL-33 is initially recognized as potent stimulator of adaptive immune response polarization toward T helper (Th) 2 phenotype following infection or exposure to allergens [13, 14]. However, the expression of IL-33 is strongly upregulated following pro-inflammatory stimuli [15]. As an alarmin released by damaged or necrotic cells IL-33 amplifies innate immune response [16] and induces marked infiltration of neutrophils, macrophages, dendritic cells, and eosinophils in various organs [17].

The elevated level of soluble ST2 in patients with polymicrobial sepsis indicated the important role of IL-33/ST2 axis in sepsis pathogenesis [18–20]. It was previously reported that IL-33 attenuates sepsis by promoting neutrophil infiltration as well as improving bacterial clearance in the peritoneal cavity [20]. Moreover, ST2 deficient phagocytes have decreased microbicidal capacities during polymicrobial sepsis in mice [20, 21]. On the other hand, elevated level of IL-33 is strongly involved in prolonged immunosuppression during the recovery of sepsis which is associated with IL-10 dependent enhancement of regulatory T cell expansion [22]. Thus, it appears that IL-33/ST2 axis may be responsible for early innate immune response to infection but also for the prolonged immunoincompetence in later phases of sepsis. The aim of the study was to delineate the importance of ST2 molecule in different pathways of innate immunity in the early phase of sepsis. We show that mice lacking ST2 molecule exhibit increased clinical score and mortality rate accompanied with lower number of granulocytes in peritoneal cavity and decreased percentage of splenic immature myeloid cells as early as 12 h following CLP. Natural killer (NK) cells from ST2^−/−^ mice show lower expression of inflammatory IFN-γ and IL-17. Septic ST2^−/−^ mice exhibit decreased presence of splenic inflammatory dendritic cells. The presence of early apoptotic spleen cells, in particular CD11c^+^ cells, was increased in septic ST2^−/−^ mice. The obtained data suggest that ST2 receptor signaling is required for the control of early inflammatory response of innate immune cells in sepsis.

## Methods

### Animals

Male, 6 weeks old ST2 deficient (ST2^−/−^) mice on BALB/c background and corresponding wild type (WT) mice were used in the experiments. All mice were housed under standard laboratory conditions (22 ± 2 °C with relative humidity of 51 ± 5% and a 12-h light: 12-h dark cycle) and were administered food and water ad libitum. Twelve hours after the induction of sepsis the mice were sacrificed and peritoneal cells and spleens were isolated for further processing.

### CLP procedure

CLP procedure was performed as described previously [23]. The mice were anesthetized by injecting intraperitoneally the solution of ketamine (70 mg/kg) (Rotexmedica, Trittau, Germany) and xylazine (13 mg/kg) (Interchemie Werken De Adelaar B.V., Venray, Netherlands) and abdominal wall was shaved. After midline laparotomy, the cecum was exposed, ligated below the ileocecal valve without causing intestinal obstruction and then punctured once with a 19G needle. The mice were resuscitated by injecting subcutaneously 1 ml of sterile 0.9% saline solution and injected subcutaneously tramadol (20 mg/kg body weight) (Hemofarm A.D., Vrsac, Serbia) for postoperative analgesia. The animals were returned immediately to a cage with exposure to an infrared heating lamp of 150 W until they recover from the anesthesia.

### Assessment of the severity of sepsis

The clinical score of sepsis, which included lethargy, piloerection, tremor, periorbital exudate, respiratory distress and diarrhea, was assessed every 6 h following CLP, as previously described [20]. Each condition was scored as 1. Mice with the clinical score of ≥1 were considered to show signs of sepsis. The survival rate was monitored for 7 days following CLP.

### Isolation of peritoneal leukocytes

Peritoneal cells were collected using 5 ml of ice-cold PBS (Sigma-Aldrich, St. Louis, MO). After supplementation with 10% of fetal bovine serum (FBS), the cells were pelleted on 1500 rpm for 8 min and resuspended in complete cell culture medium (RPMI 1640 supplemented with 10% FBS) (Invitrogen, Carlsbad, CA, USA) for further analyses.

### Isolation of splenocytes

After the excision of spleens, single-cell suspensions were obtained by mechanical disrupting. Spleens were passed through a 40 μm cell strainer (BD Biosciences, San Jose, CA, USA) and resuspended in red blood cell lyses buffer (0.155 M NH_4_Cl, 0.1 mM EDTA, 10 mM KHCO_3_), vortexed and incubated for 3 min on + 4 °C. The cells were washed twice on 1500 rpm for 8 min and resuspended in complete cell culture medium (RPMI 1640 supplemented with 10% FBS; Invitrogen) for further processing.

### Flow cytometry

Peritoneal cells or splenocytes were resuspended in FACS buffer (PBS with 5 mM EDTA and 0.2% BSA) and incubated with the fluorochrome-conjugated anti-mouse CD11b, Ly6G, Siglec-F, CD117, FcεRI, F4/80, Gr-1, Ly6C, CD49b, CD3, CD11c and CD8α (BD Biosciences/Miltenyi Biotec GmbH, Bergisch Gladbach, Germany/Biolegend, San Diego, CA, USA/Invitrogen) antibodies or their respective isotype controls. For intracellular staining, cells were incubated for 5 h at 37 °C in the presence of 50 ng/ml phorbol 12-myristate 13-acetate (PMA) (Sigma-Aldrich), 1 μg/ml ionomycin (Sigma-Aldrich) and Golgi Stop (BD Biosciences). Following the incubation, cells were fixed and permeabilized using BD Cytofix/Cytoperm buffers (BD Biosciences) and labeled with anti-mouse IFN-γ, IL-17 and IL-12 (BD Biosciences/Biolegend/R&D Systems, Inc., Minneapolis, MN, USA) (Biolegend, San Diego, CA, USA) antibodies. Isotype controls were included to set gates. Expression of cell surface and intracellular antigens was analyzed with FACSCalibur Flow Cytometer (BD Biosciences). Flow cytometric analysis was conducted with FlowJo (Tree Star) and the numerical values in the dot plots denote percentage of cells within gated quadrants.

### Immunohistochemistry

For immunohistochemical analysis, paraffin-embadded sections of spleen tissue were used. Deparaffinized tissue sections were incubated with primary rabbit anti-mouse active caspase-3 antibody (Novus Biologicals, Littleton, CO, USA, #NB100–56113) and further visualized using commercial rabbit specific HRP/AEC detection IHC Kit (Abcam, Cambridge, UK). Sections were photomicrographed with a digital camera mounted on light microscope (Olympus BX51, Japan), digitized and analyzed. The caspase 3 positive cells were determined by counting at least 1000 nuclei per slide in five randomly selected fields (at magnification × 400). The data were summarized as the mean percentage of positive cells (4–5 tissues per group). The results are presented as a mean percentage of positive stained cells per field.

### Assessment of apoptosis by flow cytometry

The mononuclear cells isolated from spleen (1 × 10^5^ cells/sample) were resuspended in 1× Binding buffer (10× Binding buffer contained 0.1 M HEPES, 1.4 M NaCl, 25 mM CaCl_2_ in distilled water, pH = 7.4) and labeled with FITC-conjugated Annexin V antibody (BD Biosciences) and Propidium iodide (PI) (50 μg/ml) (Sigma-Aldrich) for 15 min on RT. For the assessment of apoptosis of different immune cells, additional staining with fluorochrome-conjugated anti-mouse CD11c, CD19, F4/80 and CD3 antibodies (BD Biosciences/Miltenyi Biotec GmbH) was performed. Further flow cytometric analysis was conducted using FACSCalibur Flow Cytometer (BD Biosciences). The data were analyzed with FlowJo (Tree Star).

### Statistical analyses

All data are presented as means ± SE. Statistical significance between groups was determined by independent T test, and where appropriate nonparametric Mann-Whitney U test. For survival rate assessment, statistical testing between samples at each time point was determined using Fisher’s exact test. Statistical significance was assumed at *p < 0.05 and ** *p* < 0.01. Statistical analyses were performed using the SPSS 20.0.

## Results

### ST2 deficiency accelerates early polymicrobial sepsis and increases mortality

**Fig. 1:**
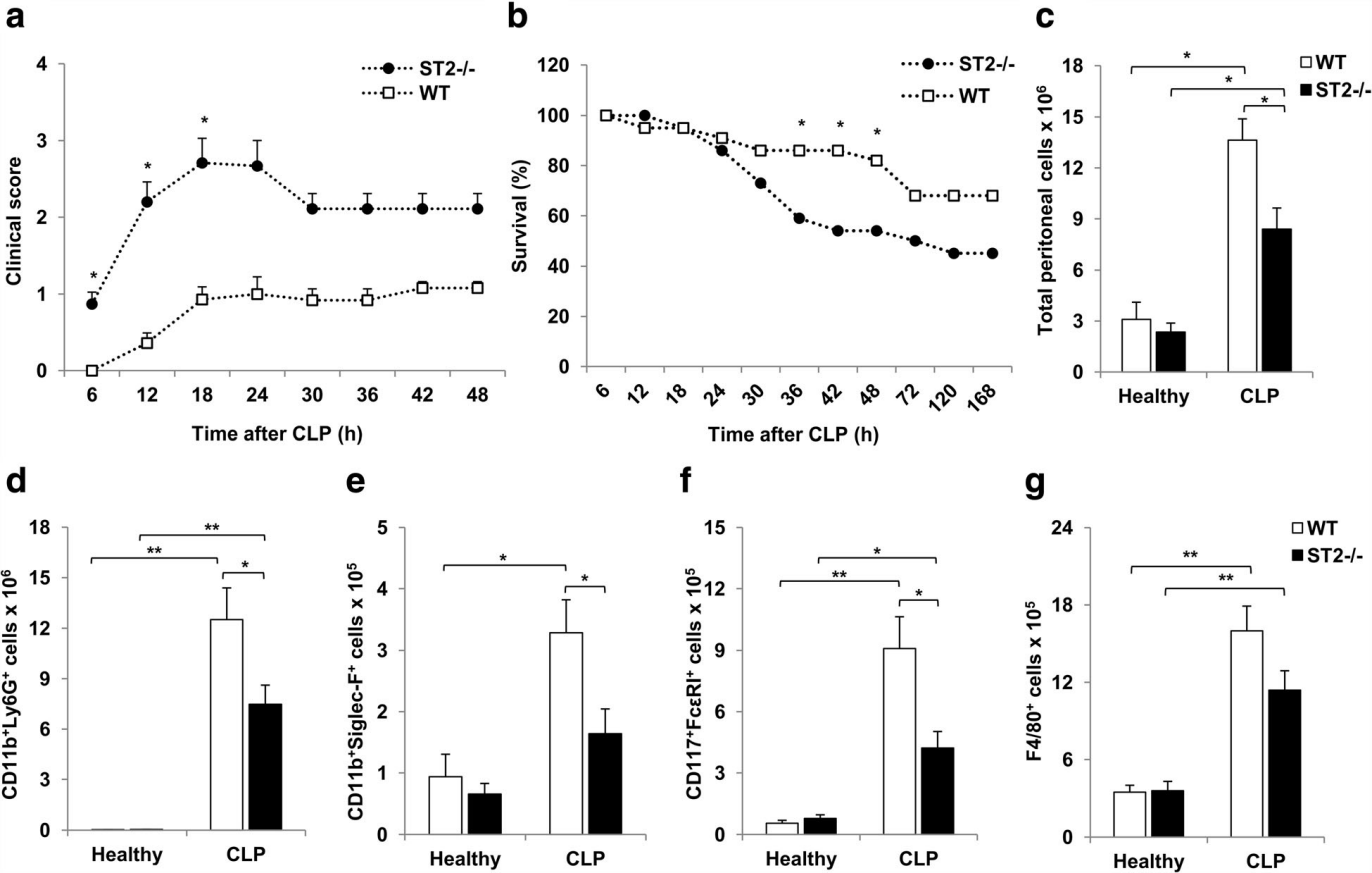
Deletion of ST2 accelerates CLP induced polymicrobial sepsis and affects granulocytes influx into peritoneal cavity. Clinical score and survival rate of septic ST2^−/−^ and WT mice was monitored every 6 h. **a** ST2^−/−^ mice exhibit significantly increased clinical score during first 18 h following CLP. **b** Survival rate of septic ST2^−/−^ and WT mice was analyzed by Fisher’s exact test for each time point. ST2^−/−^ mice had significantly decreased survival rate at 36, 42 and 48 h following CLP (*n* = 22 mice per group). **c** Significantly decreased number of peritoneal cells in septic ST2^−/−^ in comparison with WT mice 12 h following CLP. The number of peritoneal neutrophils (CD11b^+^Ly6G^+^) (**d**), eosinophils (CD11b^+^Siglec-F^+^) (**e**), mast cells (CD117^+^FcεRI^+^) (**f**) and macrophages (**g**) 12 h following CLP. Data are presented as mean ± SE, *n* = 4–7 mice per group. *p < 0.05, **p < 0.01

CLP was performed in ST2^−/−^ and WT mice and the signs of sepsis development were monitored every 6 h. The obtained data show that the clinical score in septic ST2^−/−^ mice is significantly higher compared to WT mice, in particular during first 18 h following CLP (Fig. 1a). The mortality rate of septic ST2^−/−^ mice was significantly increased 36-48 h after CLP procedure (Fig. 1b).

Systemic inflammatory response of septic mice was evaluated by serum levels of various cytokines at 12 h after CLP. The obtained data showed that CLP induces the production of IL-1β, TNF-α, IL-12, IFN-γ, IL-17 as well as IL-10, but there were no differences between two genotypes (data not shown). We also evaluated cellular make-up of peritoneal cavity. Polymicrobial challenge induced marked influx of cells into mouse peritoneal cavity (Fig. 1c). Significantly decreased number of cells was isolated from peritoneal cavity of ST2^−/−^mice in comparison with WT mice 12 h following CLP (Fig. 1c). Deletion of ST2 was strongly associated with decreased number of neutrophils (CD11b^+^Ly6G^+^) (Fig. 1d), eosinophils (CD11b^+^Siglec-F^+^) (Fig. 1e) and mast cells (CD117^+^FcεRI^+^) (Fig. 1f) in peritoneal cavity in comparison with WT mice after CLP. In addition, polymicrobial challenge markedly increased number of peritoneal macrophages (F4/80^+^), but the difference between two genotypes did not reach statistical significance 12 h after CLP (Fig. 1g).

### The number of myeloid precursor cells is lower in septic ST2^−/−^ mice

**Fig. 2:**
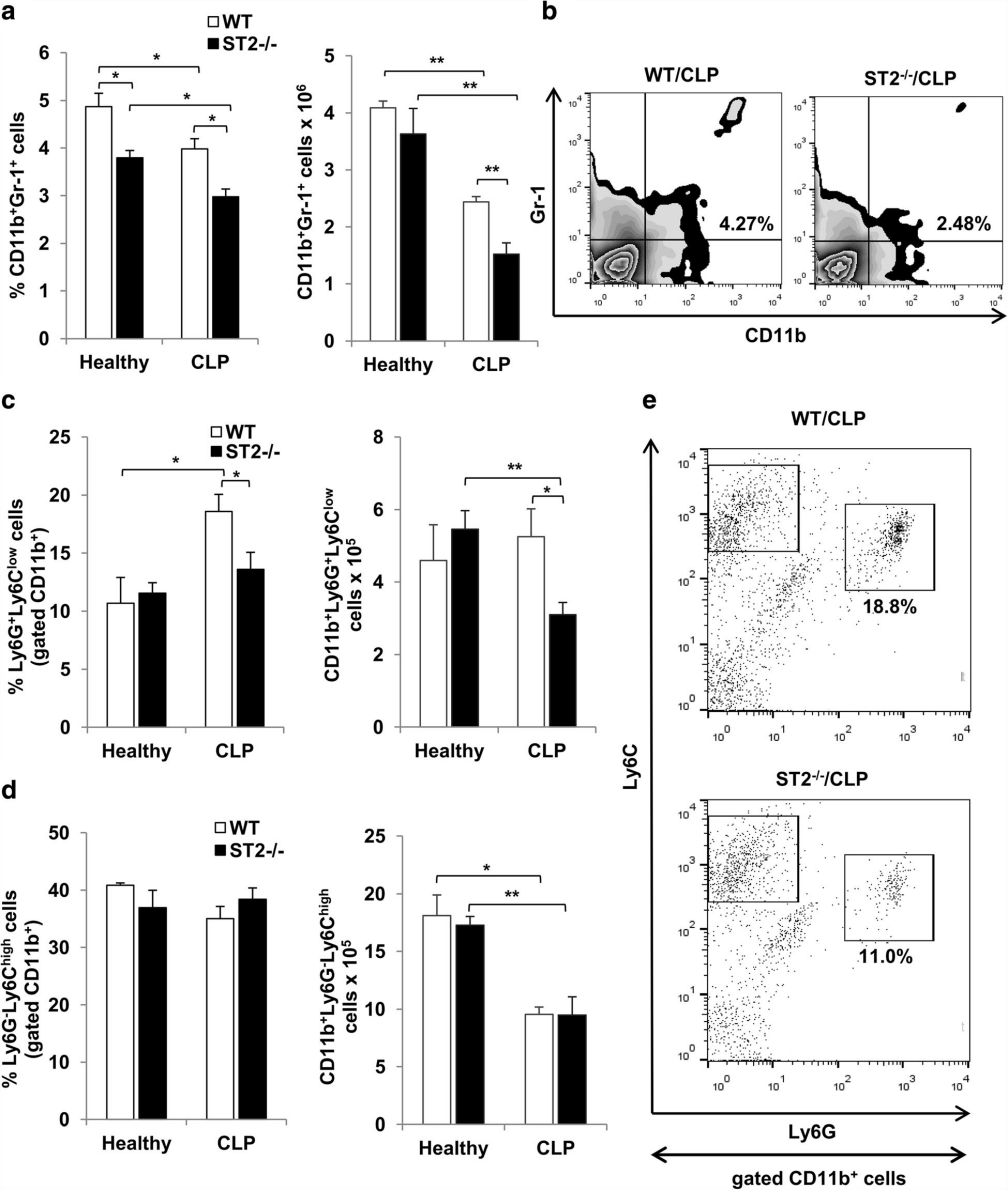
ST2 deficiency is associated with decreased number of myeloid precursors during early sepsis. **a** The frequencies and total number of splenic CD11b^+^Gr-1^+^ cells were analyzed by flow cytometry 12 h following CLP. **b** Representative plots show CD11b^+^Gr-1^+^ cells in septic ST2^−/−^ and WT mice. **c** The percentage and total number of splenic CD11b^+^Ly6G^+^Ly6C^low^ granulocytic myeloid precursors. **d** The percentage and total number of splenic CD11b^+^Ly6G^−^Ly6C^high^ monocytic myeloid precursors. **e** Representative plots denote the frequencies of granulocytic and monocytic myeloid precursors in spleen of septic ST2^−/−^ and WT mice. Data are presented as mean ± SE, *n* = 4–7 mice per group. *p < 0.05, **p < 0.01

Inflammatory response in sepsis may also depend on myeloid precursor cells which may have pro-inflammatory effects in early and suppressive effects in later phases of sepsis. As shown in Fig. 2a and b, there was lower percentage of CD11b^+^Gr-1^+^ myeloid precursors in spleen of healthy ST2^−/−^ mice, but in septic mice the difference between two genotypes reached statistical significance in both percentage and total cell number. However, septic mice of both genotypes exhibited marked decrease in percentage and total number of CD11b^+^Gr-1^+^ cells (Fig. 2a).

Under non-septic condition, there was no difference in both percentage and total number of myeloid precursor cells of granulocytic lineage (CD11b^+^Ly6G^+^Ly6C^low^) between ST2^−/−^ and WT mice (Fig. 2c). Further, polymicrobial challenge was accompanied with increased percentage of CD11b^+^Ly6G^+^Ly6C^low^ cells in WT, but not in ST2^−/−^ mice (Fig. 2c, left panel). However, the total number of CD11b^+^Ly6G^+^Ly6C^low^ cells was not affected in WT mice after CLP, while septic ST2^−/−^ mice exhibited lower number of these cells compared to healthy mice (Fig. 2c, right panel). The percentage as well as total number of myeloid precursor cells of granulocytic lineage (CD11b^+^Ly6G^+^Ly6C^low^) was significantly lower in septic ST2^−/−^ mice in comparison with WT mice (Fig. 2c and e). On the other hand, there was no difference in the percentage of myeloid precursor cells of monocytic lineage (CD11b^+^Ly6G^−^Ly6C^high^) in heathy or septic mice of both genotypes, while the total number of CD11b^+^Ly6G^−^Ly6C^high^ cells was significantly lower in septic compared to healthy mice (Fig. 2d).

### Inflammatory (IFN-γ^+^ and IL-17^+^) NK cells are decreased in septic ST2^−/−^ mice

**Fig. 3:**
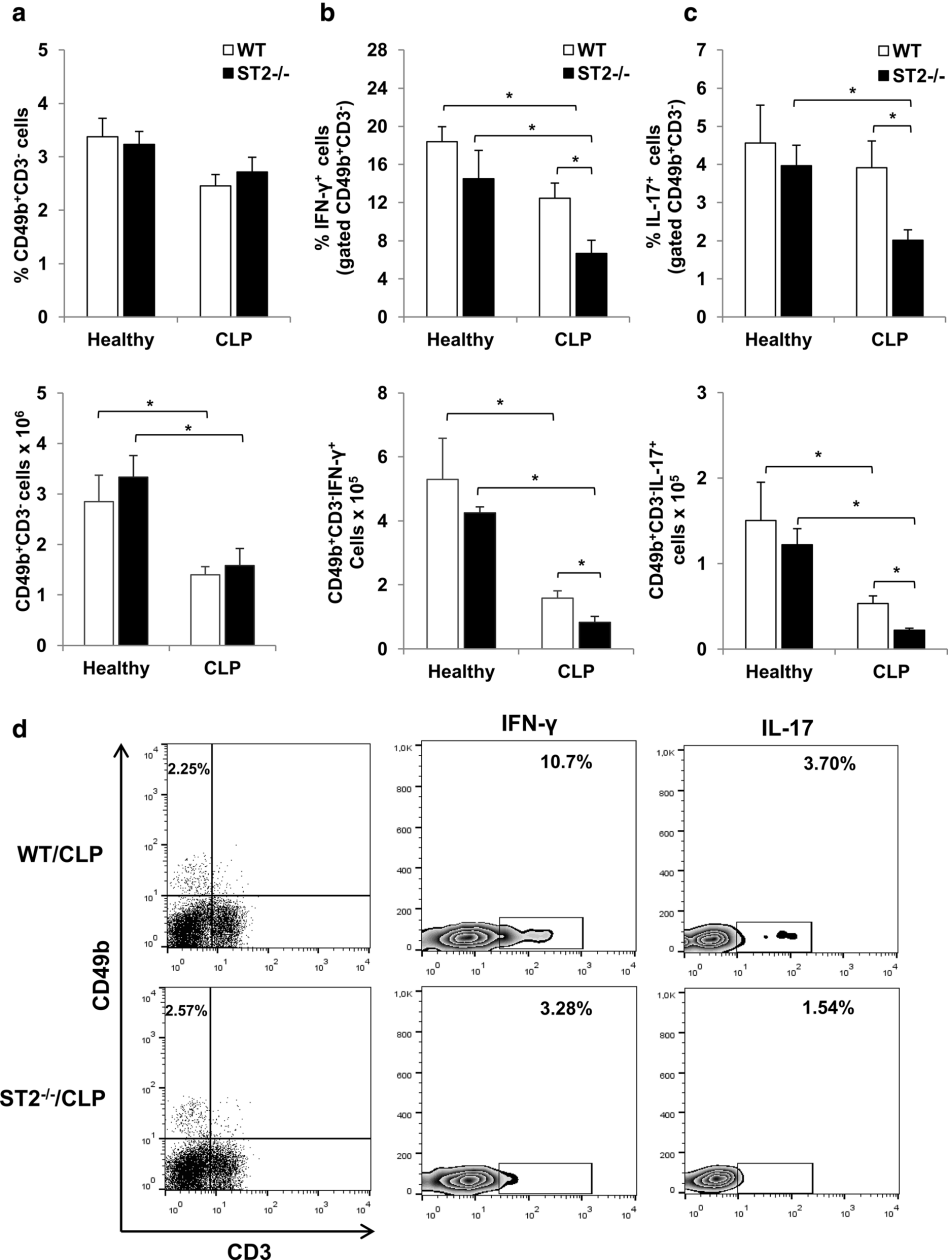
Decreased number of inflammatory NK cells in septic ST2^−/−^ mice. **a** The frequencies and total number of splenic NK (CD49b^+^CD3^−^) cells were analyzed by flow cytometry 12 h following CLP. The percentage and total number of splenic IFN-γ (**b**) and IL-17 (**c**) positive NK (CD49b^+^CD3^−^) cells. **d** The representative plots denote decreased expression of IFN-γ and IL-17 among the population of NK (CD49b^+^CD3^−^) cells derived from spleen of septic ST2^−/−^ in comparison with WT mice. Data are presented as mean ± SE, *n* = 4–5 mice per group. *p < 0.05

Apart from their cytotoxicity, NK cells are important early source of various cytokines engaged in crosstalk with other immune cell types. Although the frequencies of splenic NK (CD49b^+^CD3^−^) cells were similar between septic and healthy mice of both genotypes, the total number of NK cells was significantly decreased after CLP (Fig. 3a). Furthermore, the expression of IFN-γ and IL-17 was significantly affected in NK cells from septic mice. Significantly decreased percentage and total number of both IFN-γ and IL-17 positive NK cells was observed in ST2^−/−^ and WT mice after CLP (Fig. 3b and c). There was no difference in the expression of IFN-γ and IL-17 in NK cells between healthy ST2^−/−^ and WT mice, but in septic mice the difference between two genotypes reached statistical significance in both percentage and total cell number. In fact, in spleen of septic ST2^−/−^ mice there was significantly decreased expression of IFN-γ (Fig. 3b and d) and IL-17 (Fig. 3c and d) among NK cells in comparison with WT mice.

### Inflammatory dendritic cells are decreased in septic ST2^−/−^ mice

**Fig. 4:**
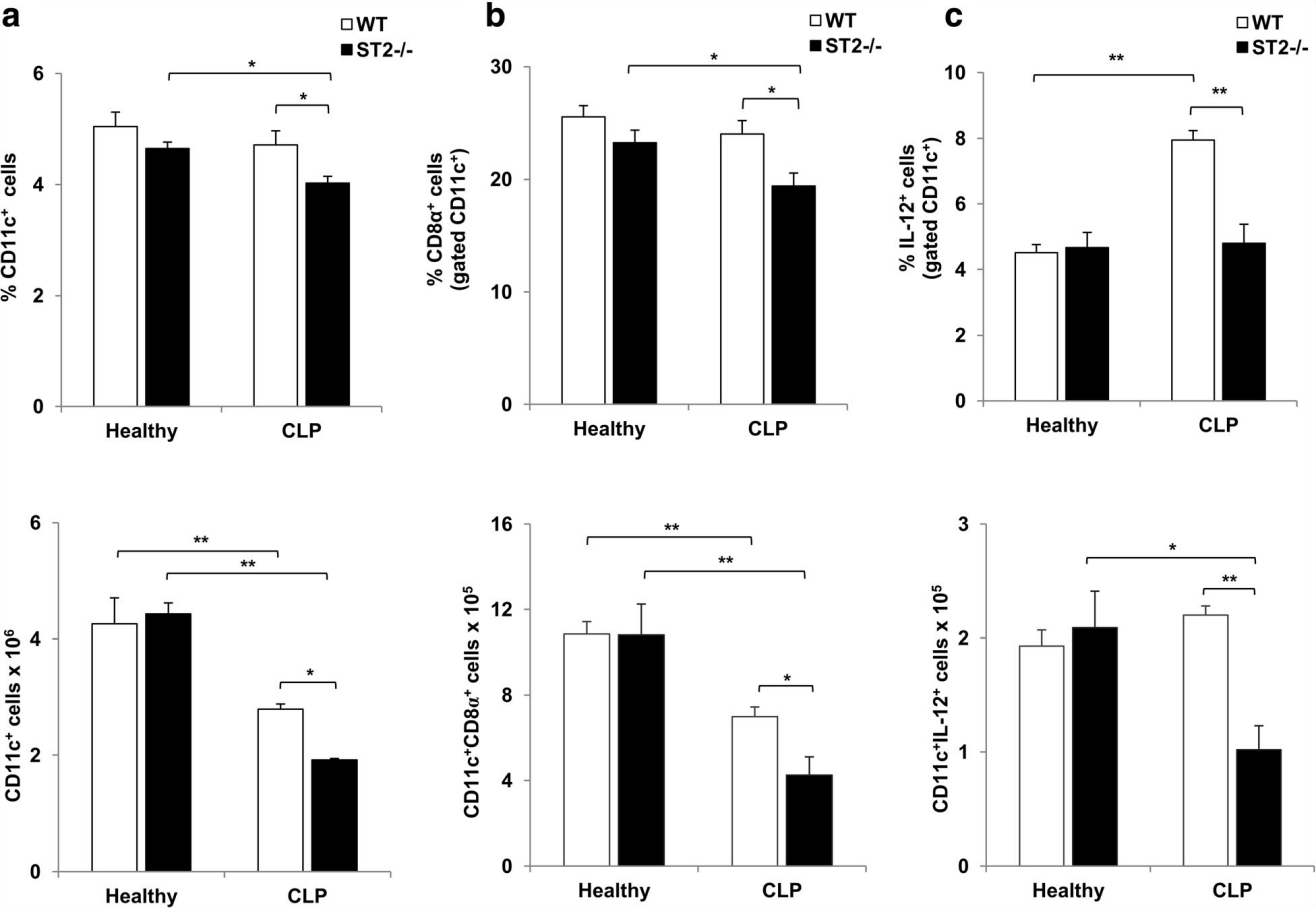
ST2 deficiency is associated with decreased number of inflammatory dendritic cells during early sepsis. **a** The percentage and total number of splenic CD11c^+^ dendritic cells was determined by flow cytometry 12 h following CLP. **b** The percentage and total number of CD8α^+^ splenic CD11c^+^ dendritic cells. **c** The percentage and total number of IL-12 expressing CD11c^+^ splenic dendritic cells. Data are presented as mean ± SE, *n* = 4–7 mice per group. *p < 0.05, **p < 0.01

In order to further delineate early innate immune response in septic ST2^−/−^ and WT mice, both presence and functional phenotype of splenic dendritic cells were analyzed 12 h after CLP. Initially, it was observed that there was no difference in the percentage and total number of dendritic cells (CD11c^+^) between healthy ST2^−/−^ and WT mice (Fig. 4a). CLP significantly decreased the total number of CD11c^+^ cells in WT mice, while in ST2^−/−^ mice the difference reached statistical significance in both percentage and total number (Fig. 4a). Twelve hours following CLP the percentage and total number of CD11c^+^ cells was significantly lower in septic ST2^−/−^ mice in comparison with WT mice (Fig. 4a). Further, there was no difference in the percentage and total number of CD8α and IL-12 positive dendritic cells between healthy ST2^−/−^ and WT mice (Fig. 4b and c). However, CLP significantly decreased the total number of CD8α^+^ dendritic cells in WT mice, while in ST2^−/−^ mice the difference reached statistical significance in both percentage and total number (Fig. 4b). In septic condition, ST2^−/−^ mice exhibited decreased percentage and total number of CD8α^+^ dendritic cells compared to WT mice (Fig. 4b). The polymicrobial challenge was accompanied with increased percentage, but not total number, of IL-12 expressing CD11c^+^ cells in WT mice (Fig. 4c). On the other hand, ST2^−/−^ mice had significantly lower number of IL-12^+^ dendritic cells after CLP (Fig. 4c, lower panel). In septic condition, ST2^−/−^ mice exhibited markedly lower percentage and total number of IL-12^+^ dendritic cells compared to WT mice (Fig. 4c).

### Sepsis-induced early apoptosis of CD11c^+^ cells is enhanced in ST2^−/−^ mice

**Fig. 5:**
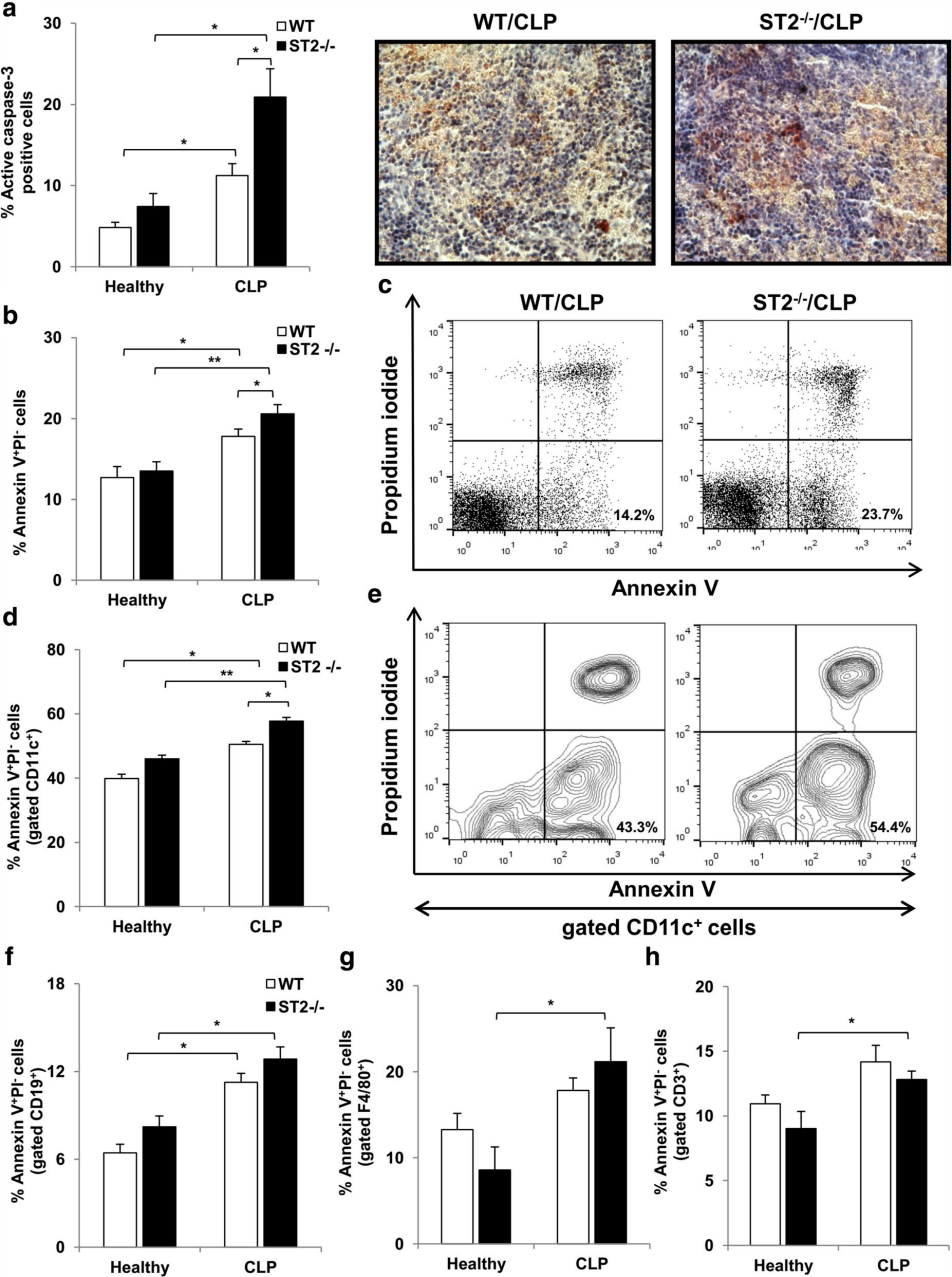
ST2 deficiency enhances early apoptosis of CD11c^+^ cells in sepsis. **a** The percentage of active caspase-3 positive spleen cells is assessed by immunohistochemistry. The results are presented as a mean percentage of positive stained nuclei per field (5 random fields, 4–5 tissues per group). Representative images show active caspase-3 positive nuclei in spleen of septic ST2^−/−^ and WT mice (magnification at × 400). **b** The percentage of early apoptotic Annexin V^+^PI^−^ spleen cells is assessed 12 h after CLP by anti-mouse Annexin V antibody and PI staining. **c** Representative plots denote the percentage of early apoptotic Annexin V^+^PI^−^ cells of total spleen cells in septic ST2^−/−^ and WT mice. **d** The percentage of early apoptotic Annexin V^+^PI^−^ spleen cells among CD11c^+^ cells. **e** Representative plots denote the percentage of early apoptotic Annexin V^+^PI^−^ cells of CD11c^+^ spleen cells in septic ST2^−/−^ and WT mice. The percentage of early apoptotic Annexin V^+^PI^−^ spleen cells among B cells (CD19^+^) (**f**), macrophages (F4/80^+^) (**g**) and T cells (CD3^+^) (**h**) was shown. Data are presented as mean ± SE, *n* = 4–7 mice per group. *p < 0.05, **p < 0.01

Immunohistochemistry showed markedly increased number of active caspase-3 positive spleen cells in septic compared to healthy mice 12 h following CLP (Fig. 5a), thus indicating higher number of cells undergoing apoptosis. Although there was no statistical difference between healthy ST2^−/−^ and WT mice, it appears that ST2^−/−^ mice exhibited significantly increased CLP-induced expression of active caspase-3 positive spleen cells compared to WT mice (Fig. 5a). In line with immunohistochemistry, sepsis is accompanied with increased percentage of early apoptotic (Annexin V^+^PI^−^) spleen cells independently of mice genotype (Fig. 5b). The frequency of Annexin V^+^PI^−^ cells was significantly increased in septic ST2^−/−^ mice in comparison with WT mice (Fig. 5b and c). In order to evaluate which immune cells undergo CLP-induced apoptosis, additional staining for membrane markers of B and T cells, dendritic cells and macrophages was performed. First, CLP increased the percentage of Annexin V^+^PI^−^ cells among CD11c^+^ population independently of mice genotype (Fig. 5d), thus indicating early apoptosis of dendritic cells. Further, ST2^−/−^ mice exhibited significantly higher frequency of CLP-induced early apoptotic CD11c^+^ cells compared to WT mice (Fig. 5d and e). CLP increased presence of early apoptotic B cells (CD19^+^) in both mice genotypes (Fig. 5f), while the percentage of Annexin V^+^PI^−^ macrophages (F4/80^+^) (Fig. 5g) and T cells (CD3^+^) (Fig. 5h) was significantly higher only in ST2^−/−^ mice. There was a trend toward increase in early apoptosis of B cells and macrophages in septic ST2^−/−^ mice in comparison with WT mice, but it did not reach statistical significance 12 h after CLP (Fig. 5f and g).

## Discussion

The present study supports the immunoprotective role of ST2 receptor signaling in the early sepsis. ST2^−/−^ mice exhibited higher clinical score as well as increased mortality rate following CLP in comparison with WT mice. Deletion of ST2 correlates with lower influx of neutrophils, eosinophils and mast cells into peritoneal cavity as well as decreased percentage of splenic myeloid precursor cells 12 h following CLP. Further, lack of ST2 contributes to decreased presence of splenic inflammatory IFN-γ^+^ and IL-17^+^ NK cells. There was lower presence of total and inflammatory dendritic cells in spleen from ST2^−/−^ mice. Higher percentage of active caspase-3 positive and early apoptotic cells, in particular CD11c^+^ cells, was observed in spleen of septic ST2^−/−^ mice.

It has been reported that multiple neutrophil dysfunctions such as impaired bacterial clearance, reduced reactive oxygen species (ROS) production and decreased recruitment to infected tissues due to loss of chemotactic activity underlies the sepsis pathogenesis [24–26]. It appears that mast cells are also participants in acute inflammatory response that release various inflammatory mediators in order to combat the infection during early stage of sepsis [27]. Accordingly, mast cells deficient mice are more susceptible to acute bacterial peritonitis [28]. Although their precise role in sepsis remains unexplored, decreased number of eosinophils correlates with increased mortality in septic patients [29]. Herein, there was marked increase in number of neutrophils, eosinophils, mast cells as well as macrophages in peritoneal cavity of both ST2^−/−^ and WT mice, 12 h after CLP (Fig. 1d, e, f and g). However, in line with previous findings [20], ST2^−/−^ mice exhibited worsen outcome in early sepsis accompanied with lower influx of neutrophils, eosinophils and mast cells (Fig. 1d, e and f). Such findings strongly correspond with the established role of IL-33 in promoting granulocytes influx [17, 30, 31] and further implicate the important role of IL-33/ST2 axis in initiating innate immune response during sepsis and subsequent efficient bacterial clearance. To these findings we here add the evidence suggesting that ST2 receptor signaling affects myeloid precursors, inflammatory NK and dendritic cells in early phase of sepsis.

Immature myeloid cells are heterogeneous cell population containing progenitors of granulocytes, macrophages or dendritic cells previously defined as C11b^+^Gr-1^+^ cells [32]. These cells are initially recognized as potent suppressors of T cells mediated anti-tumor immune response, thus termed as myeloid-derived suppressor cells (MDSCs) [33]. However, MDSCs seem to be critical participants in most acute inflammatory conditions including systemic inflammatory response during sepsis [32]. It appears that the role of CD11b^+^Gr-1^+^ MDSCs during sepsis is extremely complex and can either enhance or attenuate inflammatory response depending on the stage of sepsis progression [34, 35]. During early phase of sepsis MDSCs enhance innate immune response and express nitric oxide and proinflammatory cytokines, while in late sepsis MDSCs exhibit arginase activity and express anti-inflammatory cytokines further contributing to the protracted immunoparalysis [36]. The obtained data revealed significantly decreased presence of splenic CD11b^+^Gr-1^+^ myeloid precursors in both ST2^−/−^ and WT mice 12 h after CLP (Fig. 2a). It was previously reported that exogenous IL-33 enhances accumulation of MDSCs both in murine mammary tumor and spleen [12]. Accordingly, there was decreased percentage of CD11b^+^Gr-1^+^ myeloid precursors in spleen of ST2^−/−^ mice in both septic and healthy conditions, indicating baseline difference between two genotypes (Fig. 2a and b). However, the total number of CD11b^+^Gr-1^+^ cells did not differ between healthy ST2^−/−^ and WT mice, while septic ST2^−/−^ mice exhibited markedly lower presence of CD11b^+^Gr-1^+^ cells (Fig. 2a and b). MDSCs are subsequently divided in two different subpopulations, granulocytic Ly6G^+^Ly6C^low^ and monocytic Ly6G^−^Ly6C^high^ cells [37]. While CLP significantly increased the percentage of splenic CD11b^+^Ly6G^+^Ly6C^low^ cells of granulocytic lineage in WT mice, septic ST2^−/−^ mice exhibited lower presence of CD11b^+^Ly6G^+^Ly6C^low^ cells (Fig. 2c and e). Regarding the capabilities of MDSCs to enhance innate immune response during early sepsis, it seems that ST2 receptor signaling is important for early generation of these cells thus contributing to the development of protective immune response in sepsis.

It is well established that the early triggering of innate immune response followed by massive release of mediators of inflammation plays a critical role in sepsis pathogenesis [38]. NK cells are the integral part of innate immunity to various types of microorganisms, including viruses, bacteria, fungi or protozoa. Apart from their direct cytotoxicity, NK cells are critically involved in reciprocal interactions with other innate immune cells either directly or by cytokine secretion [39]. NK cells are main early source of IFN-γ in response to bacterial lipopolysaccharide thus indicating their pivotal role in early development of antimicrobial immunity and efficient bacterial clearance [40, 41]. Herein, sepsis was accompanied with significantly decreased number of splenic NK cells, followed by reduced expression of inflammatory IFN-γ and IL-17 (Fig. 3a, b and c). However, ST2 deficiency correlated with lower expression of IFN-γ and IL-17 among splenic NK cells following CLP (Fig. 3b, c and d). The obtained data are in accordance with evidences that IL-33/ST2 signaling enhances IFN-γ production by NK cells in IL-12 dependent manner [42, 43]. Further, it is established that IL-17 is the critical cytokine responsible for the development and mobilization of neutrophils to sites of inflammation, as well as promotion of their survival [44]. NK cells are the important source of IL-17 following certain infections such as toxoplasmosis [45]. The obtained data indicate that ST2 dependent NK cells production of inflammatory IFN-γ and IL-17 might be important for early induction of antibacterial immunity in sepsis.

As a central link between innate and adaptive immune response, CD11c positive dendritic cells are critically involved in sepsis pathogenesis. There are evidences that dendritic cells are required for the survival of mice following CLP [46, 47]. In accordance with such findings, the total number of splenic CD11c^+^, as well as CD11c^+^CD8α^+^ dendritic cells was observed in septic mice of both genotypes (Fig. 4a and b). It appears that lower presence of CD11c^+^ dendritic cells is associated with worsen outcome in septic ST2^−/−^ mice (Fig. 4a). Further, ST2 deficiency is strongly associated with decreased percentage of inflammatory CD11c^+^CD8α^+^ cells as well as IL-12 expressing CD11c^+^ dendritic cells during sepsis (Fig. 4b and c). Inflammatory CD11c^+^CD8α^+^ dendritic cells are main producers of Th1 polarizing IL-12 and IFN-γ [48, 49]. CD11c^+^CD8α^+^ cells exhibit mature dendritic cells phenotype and infiltrate cervical lymph nodes and lungs during early respiratory infection with *Bordetella pertussis* [50]. Moreover, early depletion of CD8α^+^ cells during infection is strongly associated with reduced bacterial clearance [50]. Given the fact that IL-33 activates dendritic cells during antigen presentation and promotes their recruitment [51], our data implicate the crucial role of ST2 receptor signaling in dendritic cells maturation and subsequent development of protective immune response in sepsis. Additionally, there are evidences that reciprocal interactions through direct contact or soluble mediators results in activation and cytokine production by both NK and dendritic cells [52, 53]. Herein, ST2 deficiency is accompanied with decreased presence of inflammatory dendritic cells as well as IFN-γ and IL-17 producing NK cells (Figs. 3 and 4).

Early apoptosis of lymphocytes, but also the other immune cells including macrophages and dendritic cells, is one of the central events that contributed to immune dysregulation during sepsis [54, 55]. Herein, significant increase in immune cells apoptosis was noticed in septic mice as evaluated by higher presence of active caspase-3 positive nuclei as well as early apoptotic Annexin V^+^PI^−^ cells (Fig. 5a and b). Recently, IL-33 was recognized as an important protector of cell survival [56–58]. In addition, it has been reported that exogenous IL-33 exhibits immunoprotective role in polymicrobial sepsis in mice by preventing early loss of T and B lymphocytes [59]. Our data show enhanced immune cells apoptosis in spleen of septic ST2^−/−^ mice compared to WT mice (Fig. 5a, b and c). Accordingly, there was a trend toward increase in early apoptosis of B cells and macrophages in septic ST2^−/−^ mice in comparison with WT mice, but it did not reach statistical significance 12 h after CLP (Fig. 5f and g). Interestingly, the lack of ST2 is significantly associated with CLP-induced early apoptosis of CD11c^+^ cells, indicating the loss of dendritic cells (Fig. 5d and e). The early loss of dendritic cells from secondary lymphoid organs during polymicrobial sepsis strongly predicts fatal outcome in both mice and humans [60–62]. Although CD11c is a typical dendritic cell marker, it is also possible that significant number of other cells, such as recently described CD11c^+^T-bet^+^ B cells, might contribute to high percentage of early apoptotic CD11c^+^ cells [63]. These cells are also found to be prone to cell death. However, these data implicate the significant role of ST2 receptor signaling in preventing early dendritic cells apoptosis, thus contributing to effective inflammatory response in sepsis.

## Conclusion

Taken together, the obtained data reveal that ST2 receptor signaling contributes to early development of antimicrobial immunity during sepsis. It appears that in addition to affecting influx of granulocytes, lack of ST2 profoundly alters other components of inflammatory response including myeloid precursor cells, NK and dendritic cells.

## Abbreviations

CD: Cluster of differentiation
CLP: Cecal ligation and puncture
FBS: Fetal bovine serum
FcεRI: High-affinity receptor for IgE
IFN-γ: Interferon-γ
IL: Interleukin
MDSCs: Myeloid-derived suppressor cells
NK: Natural killer
PBS: Phosphate buffered saline
PI: Propidium iodide
Th: T helper
WT: Wild type
